# In Silico Prediction of Protein Adsorption Energy on Titanium Dioxide and Gold Nanoparticles

**DOI:** 10.3390/nano10101967

**Published:** 2020-10-04

**Authors:** Shada A. Alsharif, David Power, Ian Rouse, Vladimir Lobaskin

**Affiliations:** School of Physics, University College Dublin, Belfield, Dublin 4, Ireland; shada.alsharif@ucdconnect.ie (S.A.A.); david.power.2@ucdconnect.ie (D.P.); ian.rouse@ucd.ie (I.R.)

**Keywords:** nanoparticle, protein adsorption, proteins 3D structures, Bio-nano interface, multiscale modelling

## Abstract

The free energy of adsorption of proteins onto nanoparticles offers an insight into the biological activity of these particles in the body, but calculating these energies is challenging at the atomistic resolution. In addition, structural information of the proteins may not be readily available. In this work, we demonstrate how information about adsorption affinity of proteins onto nanoparticles can be obtained from first principles with minimum experimental input. We use a multiscale model of protein–nanoparticle interaction to evaluate adsorption energies for a set of 59 human blood serum proteins on gold and titanium dioxide (anatase) nanoparticles of various sizes. For each protein, we compare the results for 3D structures derived from experiments to those predicted computationally from amino acid sequences using the I-TASSER methodology and software. Based on these calculations and 2D and 3D protein descriptors, we develop statistical models for predicting the binding energy of proteins, enabling the rapid characterization of the affinity of nanoparticles to a wide range of proteins.

## 1. Introduction

As the growth of nanotechnology accelerates, it can be expected that the number of new nanomaterials with unknown properties increases day after day. In the 21st century, the interaction of humans with NPs is commonplace, with NPs used in many household chemicals like paints and lotions [1] or novel medicines [2]. Furthermore, humans are constantly exposed to NPs from unintentional sources, including exhaust fumes from internal combustion engines [3], construction sites [4] and waste processing [5]. The protection of the health of not only the affected workers but of the general population is of growing concern.

To reduce the health risks coming from such exposure, one should identify the resulting adverse outcomes [6], trace them back to the underlying bio-nano interactions [7], and then optimise the nanomaterial itself or the relevant process to avoid the properties of concern. Ideally, this process would be performed at the stage of material’s design or production. One of the essential stages of bio-nano interaction is the formation of a biomolecular shell around a NP entering the body. This shell is known as the nanoparticle (NP) protein corona and is the biological signature of the NP that can be related to the NP physicochemical properties [8]. The contents of this corona determine which host cells interact with the NP, the type of interaction that occurs, and any adverse effects [9]. The composition of the corona will depend on many factors: the kind of the biomolecular medium which the NP entered on the way, the adsorption dynamics between different species of proteins, and many other factors.

The most informative quantity that describes the NP–protein interaction is the adsorption free energy between the NP and the protein. This quantity controls whether a protein will adhere to the NP surface to form part of the corona, whether the association is reversible, and the level of denaturing experienced by the protein. All these factors contribute to the biomolecular signature of the NP. This adsorption free energy can, in principle, be calculated ab initio through molecular dynamics, but at a significant computational cost, which renders it impractical for evaluating many NP-protein pairs. To overcome this, we previously proposed a multiscale method that allows one to compute the adsorption free energy between an NP and a protein with known 3D structure, based on a united-atom (UA) coarse-graining scheme in which each amino acid (AA) is represented by a single bead [10]. The 3D structure of the folded protein is essential for this calculation as specific characteristics (charged patches or crevasses) on the protein surface can be a significant, if not dominant, component of the interaction. Unfortunately, precise 3D structure data may not always be available for proteins of importance. The conventional ways of discovering the structure include X-ray crystallography, NMR spectroscopy, and cryo-electron microscopy, but they are limited to the proteins that form crystals [11].

In general, for the majority of proteins with known AA sequences, the 3D structure is not known. However, this sequence data may be used to predict it through computational methods. If the resulting structure is sufficiently close to the experimental one, then it is reasonable to expect that the binding energy calculated for the computational structure will likewise be close to the energy calculated for the experimental structure. Conversely, if the experimental structure is not known but can be derived from the AA sequence, then this could be used to predict the binding energy for the protein provided that this computational structure is sufficiently accurate. Thus, it is of interest to compare the binding energies between the experimental and computational structures for a set of proteins for which both are available to assess whether the binding energy for a protein with only computational structure is reliable.

A further goal that can be accomplished once a set of binding energies is built up is the derivation of a model for the prediction of binding energies based quantitatively on the properties of the protein obtained from the structure, but without requiring a full calculation using e.g., the UA approach. The protein structure and AA sequence encode a large amount of information, of which only a portion is relevant to the binding energy. Thus, finding an efficient representation of the information most important to the binding of proteins to the NP surface is key to finding a predictive model. This can be represented in terms of a set of predictors, e.g., the numbers of each type of amino acid, and the mass, charge, and volume of the protein. By calculating various predictors and keeping those with a high degree of correlation to the binding energies, a model can then be built to enable the prediction of the latter from the former.

In this work, we calculate the binding energies for 59 human blood serum proteins with the UA method using both their experimental structures and the structures predicted using the I-TASSER methodology and software (I-TASSER v5.1, Zhang group, University of Michigan) [12]. Our results indicate that these energies are sufficiently highly correlated so that the binding energy may be reasonably accurately predicted from an AA sequence in the absence of an experimental structure. Building on this, we employ the computed binding energies together with a set of predictors obtained from the protein structure to investigate the direct prediction of the binding energy using a neural network approach. We find that this neural network approach is capable of successfully predicting the binding energies of the proteins considered here on both of the nanomaterials considered over a range of radii.

## 2. Materials and Methods

### 2.1. Nanoparticles

We studied protein adsorption on NPs of two chemistries: Au and TiO_2_, which together represent the most common nanomaterials in various applications. The calculations were performed for four different spherical NPs of radii: 5 nm, 50 nm, 100 nm, and 200 nm. The physicochemical properties of each material can be found in Table 1.

### 2.2. Proteins

A list of all human blood proteins with a concentration in the serum above 10 ng/mL was obtained from the literature [13]. This list was parsed through the Research Collaboratory for Structural Bioinformatics (RCSB) mapping tool [14] to convert the UniProtIds obtained from the list into the relevant entry in the Protein DataBank (PDB) [11]. Most UniProtIds matched to more than one entry in this database due to crystallographers studying variations of the protein with different mutations, other molecules present as a complex, or different sections of the protein. A manual review of each set of PDB structures associated with a given UniProt ID was performed to select the structure with the greatest coverage of the AA sequence and as few ligands or complexed molecules as possible to ensure that the protein structure was close to that which can be predicted directly from the sequence. The exact function of a specific protein was irrelevant for this study.

To generate structures directly from the AA sequence, Iterative Threading Assembly Refinement (I-TASSER) version 5.1 was used [12]. In brief, I-TASSER is a bioinformatics method that estimates a 3D structure model of protein molecules through AA sequences and is described in more detail elsewhere [15,16]. The input for I-TASSER is a FASTA sequence file, which for this work was obtained by downloading the FASTA sequence provided by the RCSB PDB site for each PDB file. As I-TASSER is currently unable to distinguish between different chains within a FASTA sequence input, structures were calculated only for proteins with a single chain and experimental structures with multiple chains removed from the study. I-TASSER often generates more than one structure file for each protein, as many possible sequence-matches can be found. Each model produced by I-TASSER is given a confidence score (c-score). This c-score ranges from −5 to +2, where the negative values indicate that I-TASSER believes it has predicted a poor-quality 3D structure, which is unlikely to match reality. Here, we simply select the model with the highest c-score.

Ideally, the PDB structure would contain precisely one set of co-ordinates for each residue present in the AA sequence. However, due to the limits of experimental resolution and the residual thermal motion of AA side chains, this is not always possible, and PDB files may provide multiple possible locations for a single residue or indeed omit a residue (or a sequence of residues) entirely. The former is indicated using the occupancy field in the entry for a residue, which lists the probability for a given residue to be in the location stated. If the residue cannot be localised at all, the entry is simply missing, although the residue itself still contributes to the overall structure of the PDB. Structures obtained using I-TASSER, on the other hand, do not suffer from these issues and all AAs present in the sequence will be assigned a location in the final predicted structure. Thus, it may appear that an I-TASSER structure for a given protein has a different number of residues present to the PDB structure for the same protein. To take this into account, we also produce a “masked” version of each I-TASSER structure in which residues that cannot be located in the PDB structure are hidden to produce a computational structure with the same residues present as the PDB structure. This masking is performed using the sequence alignment tools provided in the BioPython library [17], which is further used to calculate the root mean square deviation (RMSD) between the PDB and (masked) I-TASSER structures. Finally, we note that the replacement of methionine by selenomethionine is not taken into account when processing PDB files and generating I-TASSER structures, as this is a random, infrequent event. The proteins considered in this study therefore consist of only the 20 canonical amino acids in their standard states.

### 2.3. Protein Descriptors

Previous studies demonstrated that statistics of adsorbed proteins and their weighted properties are predictive of a NP’s biological activity [9]. Obviously, the type of proteins the NP predominantly adsorbs reflects its own properties responsible for bio-nano interactions. For the sequence-derived descriptors, we used the pepstat program of the European Molecular Biology Open Software Suite (EMBOSS, v6.6.0, EMBOSS group, international) [18]. Several additional 3D descriptors were obtained from the protein structures, ranging from geometrical properties such as the surface area, volume, and sphericity of the protein to chemical identifiers such as the numbers of different types of amino acids on the surface. In the latter case, we evaluate both counts of individual types of AAs and of groups such as charged or aromatic AAs. A full description of the predictors is provided in Appendix A.

### 2.4. Adsorption Free Energy Calculation

The UA method was used to calculate the interaction energy between each protein and a spherical NP. A full theoretical description of the methodology can be found in [10,19,20] and all Supplementary Materials for this article are available online. The multiscale modelling performs tiered summation of the pairwise interactions between amino acids of the protein and the nanomaterial, treating each AA as a single bead located at the α-carbon position. The position of the protein relative to the NP is determined by the distance h between the centre-of-mass of the protein and the surface of the NP, and a pair of angles ϕ,θ defining the orientation of the protein. From these and the structure of the protein, the distance of each AA bead from the centre of the NP can be calculated, with the distance for bead i denoted as $r_{i}\left(h,\phi ,\theta \right)=r_{i}$. Each AA–NP interaction is computed from three contributions: a long-range term arising from the van der Waals force acting between the bulk of the NP and the AA, a short-range surface potential computed through atomistic simulations, and an electrostatic (screened Coulomb) interaction. Each of these interactions depends on factors such as the density of the nanomaterial, charged patches on the surface of the protein and the size of the NP. Here, we calculate binding energies only for spherical NPs defined by their radius *R* and neglect the electrostatic interaction. We approximate the long-range interaction between an AA bead and the NP through the Hamaker potential obtained by integration over the volumes of the NP and AA bead,

(1) $$U_{c}\left(R,R_{AA},D>r_{c}\right)=\frac{-A_{123}}{12}\left(\frac{4RR_{AA}}{D^{2}-{\left(R+R_{AA}\right)}^{2}}+\frac{4RR_{AA}}{D^{2}-{\left(R-R_{AA}\right)}^{2}}+2\ln\left(\frac{D^{2}-{\left(R-R_{AA}\right)}^{2}}{D^{2}-{\left(R+R_{AA}\right)}^{2}}\right)\right).$$

where $R_{AA}$ is the radius of the AA bead, calculated as discussed in Appendix B, D is the distance between the centre of the AA bead and the centre of the NP, $A_{123}$ is the Hamaker constant for interaction of the nanomaterial with the AA through water calculated as described in Appendix B and in previous work [10,21], and $r_{c}$ is the cutoff range for the surface potential of mean force (PMF) [10]. At short range (i.e., at distances less than $r_{c}$), part of the volume of the NP included in the above expression is already accounted for in the surface potential. We thus correct this expression by subtracting the potential for the lens segment formed by the region of the NP included in the short-range surface potential, resulting in [10],

(2) $$U_{c}\left(R,R_{AA},D<r_{c}\right)\approx U_{c}\left(R,R_{AA},D>r_{c}\right)+\frac{A_{123}}{12}\left(\frac{4\pi^{2}R_{AA}^{3}}{3D}\left(\frac{D-3R}{{\left(D-R\right)}^{3}}+\frac{-6r_{c}^{2}+8r_{c}D-3h\left(D+R\right)}{r_{c}^{4}}\right)\right).$$

The total core potential is then obtained by summing over the potential for AA beads present in the protein, $U_{c}\left(r,\phi ,\theta ,R\right)=\sum_{i}\alpha_{i}U_{c,i}\left(r_{i},R\right)$, where $\alpha_{i}$ is the occupancy of the AA bead as extracted from the PDB file. Likewise, the short-range potential is calculated through summation of the short-range PMF for each bead, $U_{s}\left(r,\phi ,\theta ,R\right)=\sum_{i}\alpha_{i}U_{s,i}\left(r_{i},R\right)$ [10,20,21]. The total potential energy U as a function of the NP-protein distance, radius of the NP and orientation of the protein is then given by,

(3) $$U\left(r,\phi ,\theta \right)=U_{s}\left(r,\phi ,\theta ,R\right)+U_{c}\left(r,\phi ,\theta ,R\right).$$

In the UA methodology, the adsorption free energy for a fixed orientation is evaluated from this expression, then a Boltzmann-weighted average over all orientations is performed to obtain the adsorption free energy [10,20,21].

Surface adsorption calculations were performed between all the proteins in the list (both the experimentally-derived and computationally-derived PDB structures) on two different nanomaterials: Au and TiO_2_. The calculations were performed for four different NP radii: 5 nm, 50 nm, 100 nm, and 200 nm. The physicochemical properties of each material can be found in Table 1. The PMFs used to define the surface contribution to the binding energy for TiO_2_ are calculated as described in [21] and for Au were calculated and reported in [10] and also presented in the Supplementary Materials, Figures S1 and S2.

### 2.5. Adsorption Affinity Ranking

To assign a numeric value to the accuracy of the affinity rankings obtained from computational structures relative to the experimental structures, we calculate the Kendall tau distance between these two lists and normalise this value to obtain the Kendall tau coefficient [22]

(4) $$\tau_{K}=1-\frac{4\;d_{K}}{N\left(N-1\right)},$$

where *d_K_* is the number of times adjacent elements in list B that must be swapped to produce the same ordering as list A, and *N* is the number of entries in the list. Under this normalization convention, the coefficient is equal to 1 if lists A and B are originally in the same ordering and –1 if one is in reverse order compared to the other. A coefficient of 0 indicates no correlation between the ordering of the two lists.

### 2.6. Prediction of Adsoprtion Energy from Protein and NP Descriptors

A further goal is the prediction of the binding energy of a protein to an NP directly from physical properties of the protein, without requiring the calculation of the binding energy via UA or some other method. To do so, we require both a training set of known sets of values of predictors and binding energies, and a model to link the two together. The simplest such model would be a linear model, in which each of these predictors is weighted by some coefficient and the sum of these weighted predictors gives the binding energy. A key disadvantage of linear models is that they are typically relevant only over a narrow range of parameters and become increasingly inaccurate outside this range. Consider, for example, the binding energy of a protein to an NP of radius *R*. It is reasonable to assume that generally this binding energy becomes larger in magnitude as *R* increases, but for very large values of *R* most of the additional NP volume is sufficiently far from the protein that it contributes very little to the binding, and thus the binding energy should saturate for sufficiently large *R*. Conversely, a linear model would simply predict that the binding scales indefinitely as the NP becomes larger. Likewise, the same argument applies for why the binding energy should not scale indefinitely as the protein grows larger for a fixed size of NP. Clearly, identifying a suitable non-linear function to describe the binding energy is a non-trivial problem. Thus, we turn to a machine learning approach and train an artificial neural network (ANN) to predict the binding energy based on the protein predictors and nanomaterial properties.

Given the very large set of protein predictors, overfitting of the ANN is a potential issue. Many of these predictors are correlated with each other, for example, a protein with many residues of a specific type will typically have a higher molecular weight. We therefore first apply a principle components analysis (PCA) to obtain a smaller set of protein predictors. The PCA is a linear transformation of the original predictors to a set of new predictors with two useful properties. Firstly, each of these predictors are uncorrelated from each other, and secondly each are associated with a value describing how much of the variance of the original predictors they describe. Thus, by selecting the PCA predictors that describe the most variance of the original predictors and discarding the rest, we obtain a more efficient representation of the proteins. We perform the PCA on the *z*-scores obtained for each predictor, that is, each predictor is normalised to have zero mean and a standard deviation of 1.

We represent the NP size in terms of ln(R) and a simple categorical value 0 for gold and 1.0 for titania. We divide the binding energies by the average Hamaker constant for the material in question to produce a dimensionless value with similar ranges for both gold and titania. This average Hamaker constant is obtained by averaging over all the Hamaker constants for the NP-AA interactions with weights given by the Dayhoff statistic $w_{AA}$ for that AA,

(5) $$\langle A_{\mathrm{NP}}\rangle =\frac{\sum_{\mathrm{AA}}A_{\mathrm{NP},\mathrm{AA}}w_{\mathrm{AA}}}{\sum_{\mathrm{AA}}w_{\mathrm{AA}}}$$

In order to avoid the requirement to manually select all parameters required for the network, we rely on the automated procedures available in Mathematica 12.1 via the Predict routine, specifying only the maximum depth for the network [23]. We initially scan over the number of layers and number of PCA variables to include to optimise these values by finding the point at which there is no further improvement in the R^2^ coefficient between the input and predicted values, then use these optimised values to generate a final network, generated as follows. To allow for a validation of the final results, we randomly divide the data set of binding energies such that two-thirds is available for training of the network and the remaining third is used for final validation. To ensure we produce a robust final result and have some measure of the uncertainty in the predicted values, we employ a bootstrap aggregation method in which we train multiple networks, each using a different random sample with replacement (bootstrap sample) of the training set. A prediction for the binding energy for a given set of protein predictors is then obtained by passing the predictors to each of these networks and averaging over their results, with an uncertainty given by the standard deviation of these predictions. We employ 30 such networks to produce this ensemble, finding that increasing the number does not significantly alter the final results.

## 3. Results

### 3.1. Protein Adsorption Energies

We used the UA methodology described in [10] to compute binding energies of 59 blood serum proteins on spherical TiO_2_ and Au NPs of radii 5, 50, 100, and 200 nm, using both the experimentally-derived structures obtained from the PDB and the structures predicted using I-TASSER method, see Methods section for details. To enable a direct comparison, in this section we show only results for the I-TASSER structure with a mask applied to exclude residues that were absent from the PDB structure. Table 2 and Table 3 show the free energy values of five of these proteins for gold and titania NP respectively over a range of radii: 5, 50, 100, and 200 nm, calculated for both PDB and masked I-TASSER structures. All the results for the complete set, including unmasked I-TASSER structures, are available in the Supplementary Materials, Table S1.

The adsorption energies shown in Table 2 and Table 3 demonstrate that the two materials have different ranges for the calculated binding energies. The Au NPs have a range between −100 to $-350k_{B}T$, whereas the TiO_2_ NP show energies in the range between 0 to −50$k_{B}T$. The strongest binding energy on 50 nm gold NP was found for 2NSM protein with binding energy equal to −288$k_{B}T$ and the most weakly binding protein was 5IR3 with binding energy approximately −137$k_{B}T$. For 50 nm TiO_2_ NP, the strongest binding was found around −27$k_{B}T$ for 6JE7, and the lowest was 1GQV with a binding free energy of $-6k_{B}T$. For most proteins, the adhesion becomes stronger as the radius of the NP increases and we note that proteins with a greater number of AAs typically also bind more strongly. Both of these effects, however, saturate at sufficiently large radii or numbers of residues if only one of these is varied.

Building on this observation, an analysis of correlations between the protein descriptors and the adsorption energy allows us to single out the most important variables. For Au NPs, the most correlated descriptors are Molecular Weight, Molar Fractions of various AA types (non-polar, charged, small, tiny, aromatic), Surface Area, Volume, and Sphericity. Obviously, there is some redundancy in this list as the surface, volume and mass as well as AA counts are not completely independent of each other. It is also clear that the interaction is dominated by the van der Waals attraction, which is additive and thus increases with the increase of the protein size, although not indefinitely. For TiO_2_ NPs, the most significant variables are Surface GLU, Surface LYS, Surface Tiny, Surface Charged, Surface Acidic, and GLU Number. Here, again, we see some redundancy. The variables reflect the dominating contribution of charge–charge interactions at the NP surface.

### 3.2. Impact of Structural Error on Binding Energies

The protein structures predicted by I-TASSER are assigned a confidence score (c-score) indicating how reliable the predictions are thought to be. This c-score is known to be correlated to the deviation between experimental structures and those generated by I-TASSER. Given that the protein adsorption energy is a function of the structure, it is of interest to see the extent to which errors in the predicted structure lead to an error in the predicted energy. We analysed the relative error in the binding energy for the TiO_2_ and Au NPs for each of the proteins as a function of their c-score and the RMSD between the I-TASSER and PDB structures. We found that the relative error is typically quite low and does not show any obvious correlation to either the c-score of the predicted structure or the RMSD, indicating that the UA approach is robust against small deviations in the protein structure. The results are slightly more dispersed for the small TiO_2_ NP than the Au NP. We attribute this to the decreased relevance of the Hamaker interaction for the small TiO_2_ NP relative to the Au NP. This interaction is reasonably insensitive to the structure of the protein, and so if it is strong the binding energy is less strongly affected by alterations to the structure of the protein.

**Figure 1:**
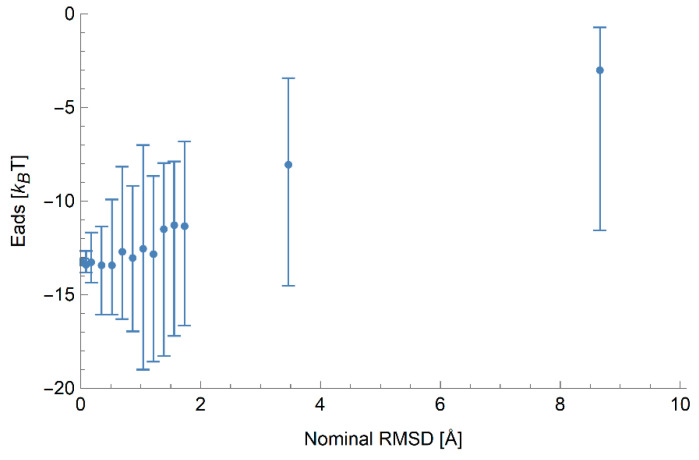
Predicted adsorption energy of 1AX8 on anatase NPs of radius 5 nm as a function of the root-mean-square deviation (RMSD) for a set of generated structures based on the PDB structure of this protein with perturbations applied to the location of each residue. The points indicate the mean binding energy of 30 structures with a specified typical RMSD, and the error bars indicate the full range of values of observed binding energies.

To further investigate the error in the binding energy caused by small errors in the location of residues, we generated an additional set of structures based on protein 1AX8. In each of these structures, the locations of the alpha carbon atoms are perturbed by small displacements drawn from a normal distribution with zero mean and standard deviation chosen to produce a known value of the RMSD relative to the initial structure. The binding energies of these structures were calculated on 5 nm anatase particles due to the high sensitivity of a small NP to the exact structure of the protein. These results are shown in Figure 1. We find that a small RMSD error of up to 1 Å does not significantly alter the mean binding energy, but does induce a large spread of binding energies, suggesting that it may be necessary to average over multiple structures if possible. As the RMSD grows larger, the spread in binding energies increases and the mean energy begins to become less strongly binding, which we attribute to the decreased density of the protein at higher RMSDs and increased likelihood of a single residue dominating the binding due to preventing the NP from making more contacts.

### 3.3. Prediction of Ranking by Binding Affinity

The UA multiscale methodology for the calculation of binding energies [10] contains several approximations, such that the output binding energy may contain inaccuracies. One of the major approximations is the assumption of rigidity of the protein 3D structure used as an input. While this approximation is crucial to enable a high throughput scanning of proteins, we would like to reduce the dependence of the result on other factors as much as possible. One specific concern is the veracity of the protein 3D structure used in the calculation. Since accurate experimental 3D structures are not known for most proteins, we must rely on a structure obtained computationally. While this necessary approximation leads to errors in the energy evaluation, we hope that the relative errors are small as some of the contributions to the interaction do not depend on the detail of 3D structure. Moreover, the approximations are likely to affect most proteins equally, such that the ranking of proteins by affinity to the NP produced by UA is likely a reasonable estimate of the true ranking. In Table 4, we give the 10 most strongly binding proteins for gold and titania NPs of radius 200 nm ordered in terms of their affinity to the NP for both the experimental and computational (masked) structures. In general, although some proteins appear in similar positions between the two lists for a given NP, overall there is not a perfect agreement. Nonetheless, it can be seen that e.g., the experimental structure most strongly binding to anatase NPs takes second place in terms of the I-TASSER structures. Given the results shown in Figure 1, it is reasonable to expect some degree of error due to the fact that the binding energies are relatively sensitive to perturbations in the structure of the protein, especially if this typical error is larger than the difference in binding energies between different proteins.

To quantify this correspondence, we calculate the normalised Kendall tau distance between the rankings of all proteins obtained for the experimental and computational structures for each of the eight NPs, as calculated using Equation (4) and presented in Table 5. The coefficients obtained are typically on the order of 0.6, indicating that although there is a correlation between the binding energies obtained for experimental and computational structures, the exact ranking may differ. A clear outlier is the lower value of 0.48 for the 5 nm gold NP, which we attribute to the increased significance in minor variations of the protein structure for binding to small NPs as this may alter which residues are in close-contact with the NP. A similar effect is observed to a lesser extent for the TiO_2_ NP, providing further confirmation that the poorer agreement is due to the combination of size of the NP with the size of the specific cavities on the rigid protein globule. Overall for Au 200 nm NP, 7 out of 10 strongest binding proteins coincide for the two methods, for TiO_2_ 200 nm NP 5 out of top 10 are the same.

**Figure 2:**
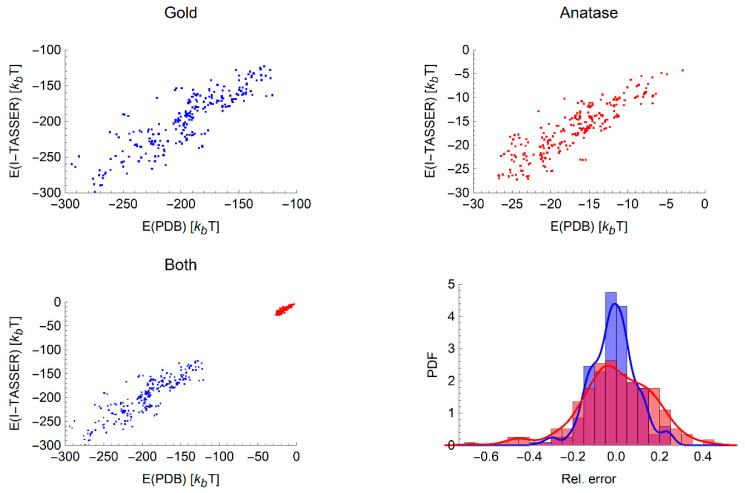
Comparison between adsorption free energies for the experimentally and computationally derived protein structures, showing all radii simultaneously for gold (**top left**), anatase (**top right**) and the combined data sets (**bottom left**). **Bottom right:** The sets of relative error of the two materials with blue bars indicating the data for gold and orange for anatase presented as probability density functions (PDF), i.e., normalised counts. A smoothed line (blue for gold, red for anatase) is added to guide the eye and distinguish between overlapping regions.

Having confirmed that the ranking of proteins by the binding affinity is mostly consistent between the experimental and computational structures, we next turn to evaluating if there is a simple relationship between the values of the binding energies obtained from computational and experimental structures. Despite the fact that there is not a one-to-one correspondence between the ranking of experimental and computational proteins for a given NP, we nonetheless observe a linear correlation between these two binding energies for both nanomaterials over all radii as shown in Figure 2. We also observe that the relative error between the binding energies for the experimental and computational structures are quite small, and the vast majority fall within ±20%. The $R^{2}$ statistics for the best-fitting linear relationship between experimental and computational binding energies are listed in Table 6 for each NP. These are again in most cases around the 0.6–0.8 range, reflecting the fact that the binding energy may vary due to relatively small changes in the structure.

### 3.4. Metamodel of Adsorption

Given the relatively high correlation between the binding energies obtained using experimental and computational structures, even when the predicted confidence in the structure is low, it is reasonable to assume that the binding energy is not highly dependent on the exact structure of the protein in general. Thus, we proceed with the construction of a model that includes the structure of the protein indirectly using the predictors discussed in Section 2.3. To enable ab initio prediction of protein binding energies, we calculate these predictors using the I-TASSER structures without masking of the proteins, that is, using the full sequence modelled, and train the network on the energies predicted using UnitedAtom for these structures. To prepare input for the neural network approach, we first perform principal component analysis (PCA) of the set of protein predictors to eliminate the high degree of redundancy between the predictors. The coefficients describing the contributions to each of the first 10 of the resulting variables from each of the protein predictors are provided in Supplementary Materials, along with the mean and standard deviation required to convert the predictor to its associated *z*-score. The proteins vary significantly in structure and composition and this is reflected in the percentages of variation captured by each PCA variable, with the most significant variable covering 30% of the variation. This procedure produces a dimensionless set of input variables, together with the material index (0 for gold, 1 for titania) and the logarithm of the radius of the NP expressed in nanometres. If a larger set of materials is available, one could replace this index by a physics material descriptor to make a universal model. We divide the binding energies by the average Hamaker constant for that material calculated using Equation (5), $\langle A_{Au}\rangle =72.1k_{B}T,\langle A_{Anatase}\rangle =8.78k_{B}T$, such that the network predicts a dimensionless variable with a similar range for both materials.

**Figure 3:**
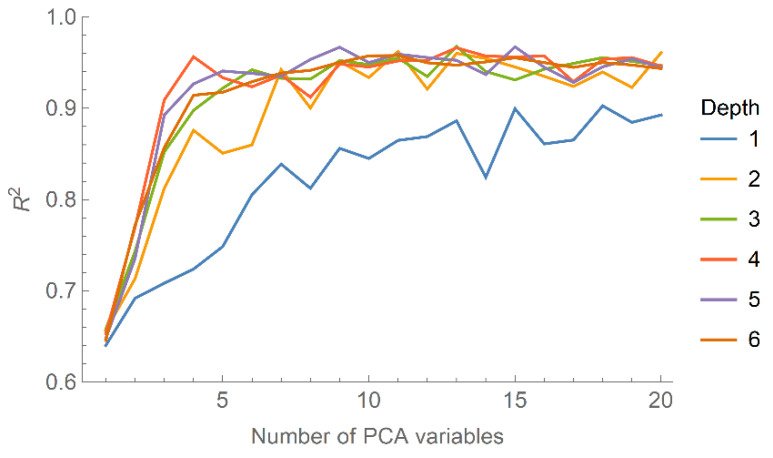
The R^2^ coefficient describing the accuracy of the predicted binding energies from the neural network as a function of the number of PCA variables included, shown for network depths ranging from 1 to 6.

To optimise the neural network with respect to the depth of the network and number of PCA variables to use as input, we perform a brute-force search over these two parameters and calculate the R^2^ coefficient between the predicted and input binding (dimensionless) energies. The results are shown in Figure 3, from which it can be seen that R^2^ effectively saturates above four layers and quite rapidly with respect to the number of PCA variables included. A network with too many free parameters, e.g., number of layers and number of variables, is prone to overfitting, limiting its ability to make meaningful predictions for variables outside the training set. Thus, we select four layers and the first three PCA variables as a trade-off between accuracy and preventing overfitting.

**Figure 4:**
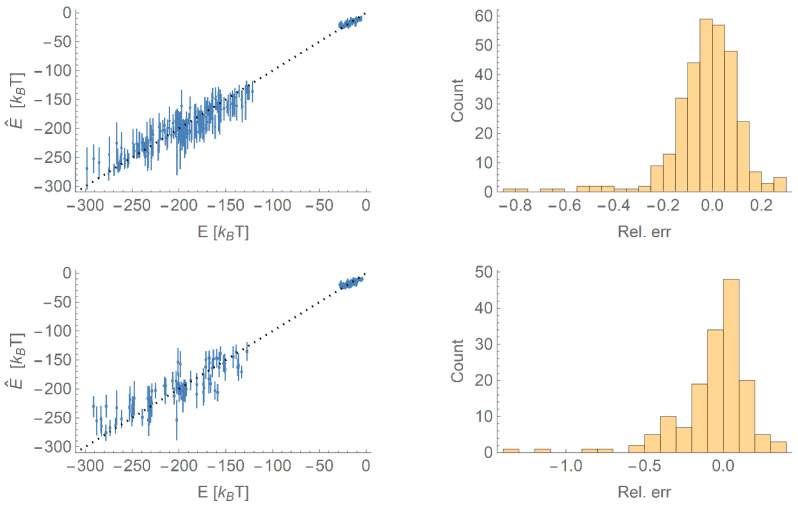
**Top**: A comparison of the binding energies predicted by the neural network ($\hat{E}$) compared to the binding energies (*E*) used as input (**left**) and a histogram of the relative errors (**right**) for data points in the training set. Error bars indicate the standard deviation in the predicted values. **Bottom**: As top, except for points in the validation set.

We then train an ensemble of 30 networks on bootstrap samples of the training set of two-thirds of the data, with the first three PCA variables, NP material and NP radius as input and the scaled binding energy as output. This training set is selected at random such that all proteins and NPs of all radii for both materials are sampled in the initial set. The results are shown in Figure 4, showing predictions for both the training set and the validation set consisting of the remaining data. For clarity and to distinguish between the TiO_2_ and Au NPs, we have rescaled the energies back from the dimensionless units used by the network to units of *k*_B_*T*. We also plot histograms of the relative errors in the binding energy for these two sets; it can be seen that there is a slight tendency to underpredict the binding energy in both training and validation sets, but overall the relative error is quite small. The agreement is worse for the validation set, as expected, but indicates that the ANN model is capable of predicting binding energies within a reasonable margin of error. In terms of the ranking of binding energies (again in the dimensionless units output from the network to enable a fair comparison between different materials), we find Kendall tau coefficients of 0.77 for the training set and 0.68 for the validation set, confirming that the ANN approach successfully predicts the correct ordering for most proteins by binding affinity to the NP.

**Figure 5:**
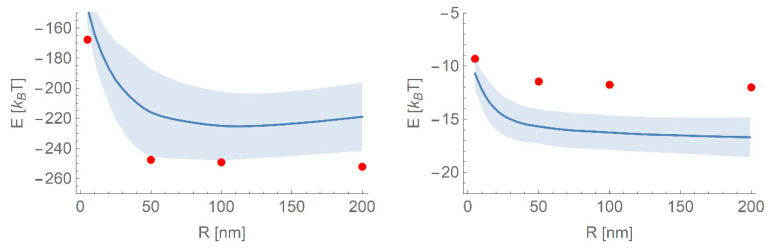
The predicted trends in binding energies as a function of the radius of the NP for gold (**left**) and TiO_2_ (**right**) for the protein with PDB ID 1AX8. The known binding energies are shown as points, and the solid line indicates the mean of the ensemble prediction for that protein at that radius. The shaded area indicates ± one standard deviation of the ensemble predictions around the given radius.

Since the network is trained on a range of NP sizes, it is of interest to see if it can successfully interpolate between these to be able to predict the binding energy of a protein to an NP of radius between 1–200 nm other than those sizes provided in the initial training set. In Figure 5, we plot the binding energy as a function of radius for a given protein (1AX8) with the binding energies at known radii shown as points. The model has produced a physically realistic interpolation in most cases, that is, the behaviour is reasonably smooth, indicating that the network is not overfitting to the data and can be used to produce a first estimate of the binding energies for a wider range of radii. In this case, however, the model fails to accurately predict the binding energy for this protein to either material within one standard deviation. This is likely due to the limited size of the training set and the simple variable used to distinguish between gold and titania NPs; with a more physically meaningful variable, it is likely that the accuracy can be increased further.

## 4. Discussion

In this paper, we computationally predict a set of 59 human blood serum proteins structures from their AA sequences using the I-TASSER software suite. The binding energies for these structures on Au and TiO_2_ NPs over a range of radii are in relatively good agreement with the values calculated for the experimental structures obtained from the PDB. From the resulting R^2^ coefficients (typically greater than 0.6), it can clearly be seen that there is a strong linear correlation between the binding energies computed for protein structures taken from the PDB and calculated using I-TASSER, with slightly better agreement found for larger NPs. We attribute this to the increased relevance of bulk van der Waals forces, which are less strongly influenced by the exact structure of the protein and depend more on its overall volume. In general, the good agreement between the binding energies obtained for computational and experimental structures, both in terms of ranking the protein–NP affinity and the linear correlation between these two energies, confirms that in the absence of an experimental structure the binding energy of a single-chain protein can be reasonably well estimated from a structure obtained using I-TASSER. Indeed, given the fact that many experimental sequences are missing residues or do not cover the entire protein, it may even be preferable to use a computational structure, or multiple structures if these are available to allow for an averaging over small errors in the placement of residues. This good agreement does not necessarily imply that the binding energy obtained using either of these two types of structures is accurate to the binding energy that could be found experimentally or using a more complex simulation. Rather, it indicates that the accuracy of the UA model is not significantly impacted by using a protein structure obtained via I-TASSER.

As mentioned earlier, both nanomaterials tend to have different ranges for the calculated binding energies as can be seen in Figure 1. The Au NPs have a range between −100 to −350*k_B_T,* whereas the TiO_2_ NPs have an energy range between 0 to −50*k_B_T*. The main reason for this is the differing strength of the total van der Waals interaction for each nanomaterial, as parameterised by the Hamaker constant, and different degree of hydrophilicity of the two materials. In the UA model employed, the Hamaker constant is calculated between each nanomaterial and AA residue and used to determine the strength of the long-range interaction. Typically, the Hamaker constant for an Au–AA interaction is on the order of 70$k_{B}T$ (174.58 kJ·mol^−1^), an order of magnitude larger than that for a TiO_2_ interaction 7$k_{B}T$ (17.458 kJ·mol^−1^). Likewise, the potentials of mean force describing the surface interaction between an NP and an AA are also typically much stronger for Au than for TiO_2_, reflecting the strong preferential water adsorption at the TiO_2_ surface. For most AA on TiO_2_, their preferred position is some distance away from the solid, allowing for a water layer to stay in between. Titania materials are known for their extreme hydrophilicity [19,21]. Regardless of the material, we find that the absolute values of the binding energies calculated for experimental and computational structures are reasonably close to each other.

The calculated binding energies can, in theory, be used for predicting the content of NP protein coronas [24,25]. However, a direct comparison of the ranking predicted from the adsorption energies to the experimentally measured protein abundances in the corona would not produce meaningful results. Firstly, the actual abundance of the protein in the corona would depend on the concentration of the specific protein in the medium. These concentrations may differ by several orders of magnitude, and the entropic contribution could dominate the adsorption rate such that a protein that is weakly binding but highly abundant in the medium could be more strongly present in the corona than a protein that is less abundant but more strongly binding. In addition, for materials like gold, the NP–protein interaction leads to irreversible adsorption, again favouring proteins that are more abundant in the medium and so are adsorbed more quickly. This leads to the requirement to generate a more advanced model of corona formation, which is currently under development.

To validate our model of binding free energies, we compared the binding energy with available experimental data. The binding energies of proteins to gold NPs have been previously experimentally measured for insulin (PDB-ID: 4INS) and liver alcohol dehydrogenase (LAHD, PDB-ID: 1HET) [26]. The calculated energy of adsorption from UA for insulin on the smallest NP in [26] and employing a Boltzmann average over orientations is −411 kJ/mol, which is higher than the experimental value reported −51.6 kJ/mol. For LAHD, the calculated adsorption energy is −428 kJ/mol and the experimental value recorded is −54.0 kJ/mol. The binding energies obtained from the UA model are very large, which is a result of very strong contact attractions we see for most AA PMFs at the surface and high van der Waals attractions. Although the approximations made in the UA model can be partly blamed for the exaggeration of the attraction, we find that the atomistic force field is responsible for most of the effect. Nevertheless, the UA model correctly predicts a stronger binding for LAHD in agreement with the experiment. We note, furthermore, that given the strength of the interaction, the adsorption must be irreversible on experimental timescales for most proteins on particles of sufficiently large size (*R* > 5 nm), which implies that this discrepancy could be caused by the inability of most experimental techniques to accurately assign an adsorption energy when the binding is essentially irreversible. If we instead employ a simple average over orientations, more closely approximating the binding of proteins before an equilibrium is reached, we obtain binding energies of −157 kJ/mol (insulin) and −153 kJ/mol (LAHD), which are closer to the experimental values but still more strongly binding.

With regards to the prediction of adsorption energy from first principles, we cannot yet apply the method to an arbitrary material. A key limitation of our neural network model is that, at present, we do not yet have a well-defined means to fully distinguish between the two materials considered. The difference in their long-range van der Waals interaction is accounted for by scaling the binding energies by the average Hamaker constant, such that the output from the model need only be multiplied by this value to obtain a material-specific binding energy. However, the short-range interactions of these materials with AAs differ significantly in terms of their relative hydrophilicities, in that gold NPs are relatively insensitive to the exact type of AA to which they bind, whereas TiO_2_ NPs are much more selective. In the current model, this is simply encoded in terms of a variable set to 0 for gold and 1 for TiO_2_. A clear goal for future work would be to perform calculations for a greater range of materials and build these results into the network along with a more physically meaningful interpretation of this variable. For example, it is possible that this variable can be replaced by the surface charge or some measure of the immersion enthalpy. With only two possible materials, such a choice is arbitrary as any such measure can be rescaled to fall in the range of 0–1 and so reduces to the variable employed here. Including more materials, however, would enable a more meaningful choice to be made, and ideally the network could then be employed to predict binding energies of NPs of a range of materials outside the training set, provided that these can be adequately represented by a Hamaker constant and the unknown surface-dependent variable. This remains outside the scope of the present paper, but the calculation of binding energies for a wide range of nanomaterials is presently underway.

Likewise, the model as presented here is limited to spherical NPs, whereas there is clear interest in other geometries e.g., cylindrical NPs as models for rod-like nanomaterials and carbon nanotubes. We have extended the UnitedAtom methodology to predict binding energies of proteins on cylindrical NPs, but the modifications required are extensive and will be reported elsewhere. Generally, we find that cylindrical geometry facilitates the protein binding as it allows more contacts with the NP for the same protein. As an example, the adhesion of the I-TASSER structure for the strongest-binding protein 6NCO onto gold rods is considerably stronger for a rod of radius 5 nm ($-321k_{B}T$) than for a sphere of the same radius ($-210k_{B}T)$. At larger radii R=200 nm, we observe binding energies of $-298k_{B}T$ for a sphere and $-306k_{B}T$ for a rod, suggesting that the main effect is due to the difference in surface curvature for the two geometries, which is less pronounced at greater radii.

Despite the above limitations, we find that the set of predictors used here is capable of training a neural network to successfully predict the binding energy of proteins on NPs of two materials and a range of sizes and appears to allow for the interpolation between these sizes, thus enabling the rapid evaluation of the protein affinity of NPs of a known material but differing size for this set of proteins. An additional goal for further work would be to refine the set of predictors and the network architecture to enable the accurate prediction of the binding energies of proteins from outside the set used for the training of the network. This, however, will likely take a much larger set of proteins for training due to the large number of predictors required to describe a protein. We stress also that although the binding energies used here have been calculated using the UA method, in principle the ANN approach could be applied equally well to predict binding energies calculated using more in-depth simulations or obtained experimentally.

## 5. Conclusions

In this work, we have calculated the binding energy of proteins to Au and TiO_2_ NPs of various sizes using a multiscale interaction model and protein structures obtained both experimentally and computationally from AA sequences. We find that the calculation based on predicted protein 3D structures provides a good approximation to the binding energy, thus it can be reliably used for cases where a protein 3D structure is not known. The binding energy range tends to be higher on Au NP surface as compared to TiO_2_ due to stronger van der Waals core interaction and closer approach of the AA to the surface atoms. The weaker attraction of the proteins to the titania NPs can be attributed to strong hydrophilicity of the surfaces, which prefer to bind water rather than AAs. For both materials, we observe that binding energies are typically greater for larger proteins and NPs. We introduced several novel protein descriptors based on their 3D structure and have developed a neural network model that predicts the protein adsorption energy from basic nanomaterial and protein descriptors, furthering the ability to estimate the affinity of a protein to a given NP when only an approximate structure can be calculated. The binding energies calculated here are of use in further studies, e.g., for the prediction of the corona content of NPs immersed in biological media, and themselves represent important quantities parameterizing the interactions of nanomaterials with biomaterials and serving as predictors of their biological activity. For example, we are presently exploring the use of binding energies calculated using this method as input for quantitative structure–activity relationship models to predict the inflammation response caused by nanomaterials. Previous examples of predictive models for NP uptake and association with live cells demonstrate that this may be indeed possible [24,25].

## Figures and Tables

**Table 1:** Characteristics of the two nanomaterials considered in this paper, detailing the parameters required for the atomistic simulations and the optical characteristics required for the calculation of Hamaker constants.

Material	Allotrope	Miller Index	n (λ = 550 nm)	$\nu_{0}$, 10^15^ Hz
TiO_2_	Anatase	101	2.5287	1.50
Au	FCC	100	0.4242	4.87

**Table 2:** Binding-free energy values of five sample proteins for their structures taken from the PDB and calculated using I-TASSER on gold NPs of radius 5, 50, 100, and 200 nm.

ID	Structure	*E*(*R* = 5 nm) [*k*_B_*T*]	*E*(*R* = 50 nm) [*k*_B_*T*]	*E*(*R* = 100 nm) [*k*_B_*T*]	*E*(*R* = 200 nm) [*k*_B_*T*]
1AX8	PDB	−152.5	−214.2	−219.4	−222.3
	I-TASSER	−168.8	−242.1	−249.0	−249.2
1F5F	PDB	−130.7	−174.1	−179.1	−180.6
	I-TASSER	−135.6	−175.3	−180.3	−182.3
1GQV	PDB	−146.0	−195.0	−201.0	−204.3
	I-TASSER	−160.9	−223.8	−228.6	−230.9
1HPT	PDB	−145.6	−158.1	−159.6	−161.5
	I-TASSER	−171.5	−178.8	−179.1	−180.2
1HUP	PDB	−131.2	−192.8	−201.3	−204.3
	I-TASSER	−126.9	−198.0	−206.4	−211.0

**Table 3:** Binding-free energy values of five sample proteins for their structures taken from the PDB and calculated using I-TASSER on for titanium dioxide NPs of radius 5, 50, 100, and 200 nm.

ID	Structure	*E*(*R* = 5 nm) [ *k*_B_*T*]	*E*(*R* = 50 nm) [*k*_B_*T*]	*E*(*R* = 100 nm) [*k*_B_*T*]	*E*(*R* = 200 nm) [*k*_B_*T*]
1AX8	PDB	−13.3	−15.0	−15.1	−15.1
	I-TASSER	−9.4	−11.6	−11.8	−11.9
1F5F	PDB	−16.1	−20.0	−20.0	−20.3
	I-TASSER	−15.5	−18.9	−18.9	−19.0
1GQV	PDB	−2.9	−6.3	−6.7	−6.8
	I-TASSER	−4.3	−9.2	−9.6	−9.7
1HPT	PDB	−7.3	−8.7	−8.9	−9.0
	I-TASSER	−7.1	−9.8	−9.9	−10.1
1HUP	PDB	−11.7	−16.8	−16.6	−16.4
	I-TASSER	−11.7	−14.1	−14.6	−14.6

**Table 4:** Ranking of proteins by affinity to the NP for both the experimental and computational structures, ordered by the binding strength for the most strongly binding proteins. (All ranking values for both NPs in all sizes are available in the attached *E*-ranking file on the supplementary section).

Ranking	Au, 200 nm		TiO_2_, 200 nm	
	Experimental Structure	I-TASSER Structure	Experimental Structure	I-TASSER Structure
1	2NSM	6NCO	3GW3	6NCO
2	3GW3	5O7D	2QYQ	3GW3
3	6NCO	3GW3	4DOU	5VHG
4	2RHP	3DHP	5VC1	4DOU
5	5O7D	2RHP	6JE7	6JE7
6	3DHP	2NSM	4GLP	2FJ9
7	5EC3	1ZXQ	1NUH	9CA2
8	4YEQ	4XAT	6M8Z	4NH9
9	4XAT	4CYY	1IMV	5EC3
10	5VC1	1NUH	6NCO	6M8Z

**Table 5:** Kendal Tau correlation coefficient between adsorption affinity rankings for the experimental and computational protein 3D structures for spherical NPs of various radii *R*.

Material	200 nm	100 nm	50 nm	5 nm
Au	0.61	0.62	0.64	0.48
TiO_2_	0.66	0.66	0.65	0.59

**Table 6:** $R^{2}$ values for a linear best fit between the experimental and computational protein 3D structures for spherical NPs of various radii *R*, allowing for both a linear and constant term.

Material	200 nm	100 nm	50 nm	5 nm
Au	0.68	0.67	0.69	0.47
TiO_2_	0.72	0.73	0.72	0.62

**Figure A1:**
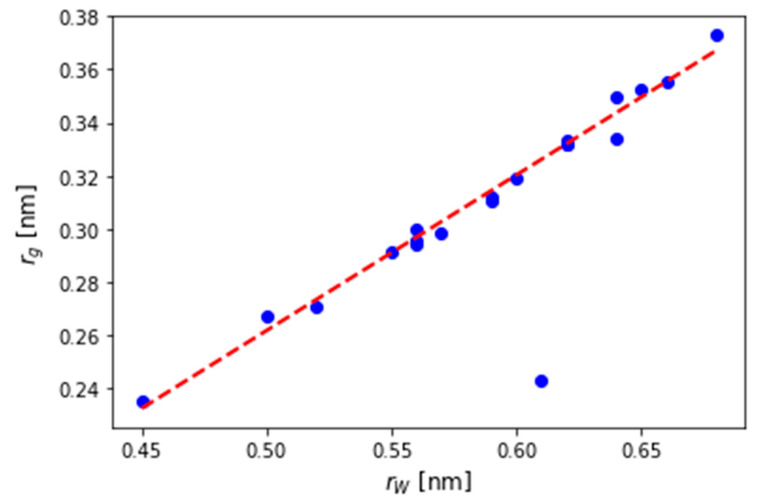
Comparison between $r_{g}$ and $r_{W}$ for the 20 amino acids. The ratio of $r_{g}$ to $r_{W}$ is similar for each AA (with HIS being the only outlier).

**Figure A2:**
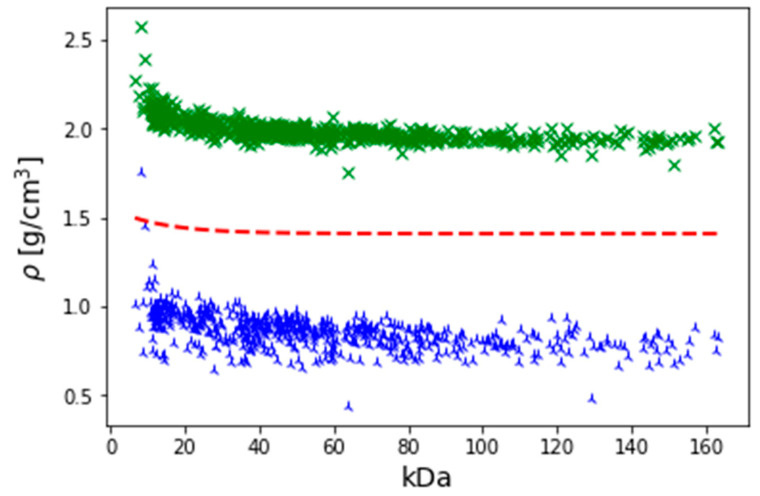
A plot of density versus mass for a range of proteins of various sizes. Blue: van der Waals radius. Green: Etch radius. Red: Experimentally desired result from Equation (A7).

**Figure A3:**
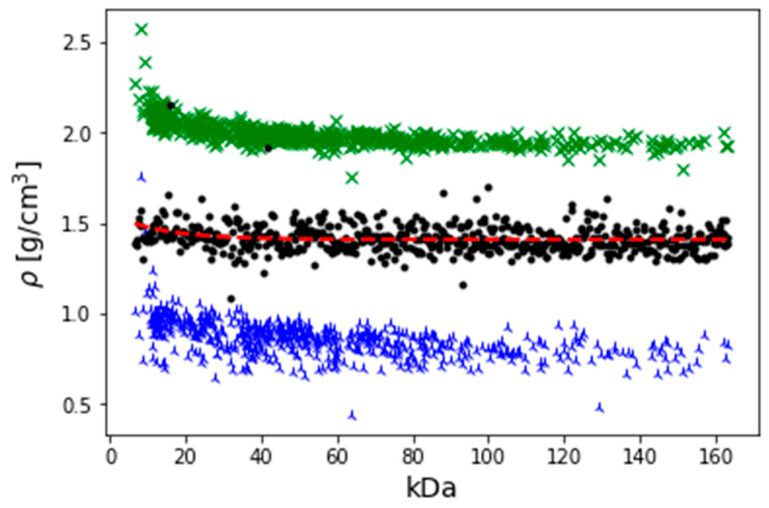
A distribution of density versus mass for a range of proteins of various sizes. Blue: van der Waals radius. Green: Etch radius Red: Experimentally desired result Black: Optimised linear combination of van der Waals radius and etch radius.

## Supplementary Materials

The following are available online at https://www.mdpi.com/2079-4991/10/10/1967/s1, Figure S1: Potentials of mean force for amino acid–TiO_2_ interaction, Figure S2: Potentials of mean force for amino acid -gold interaction, Table S1: Adsorption free energies and ranking for 59 proteins on Au and TiO_2_ NPs of four different sizes calculated for PDB structures and I-TASSER structures with and without mask, Mathematica notebook for the training of the neural network “ProteinBindingEnergyNN-59.nb” and tabulated predictors and adsorption energies used as input “ProteinPredictorSet.csv”.

## Appendix A

### Protein Descriptors

Below, we summarise the predictors calculated from protein structures for use in the metamodel in addition to those calculated using Emboss pepstats [18]:

- **Solvent-accessible surface area:** This the surface area of a protein that can be accessed by solvents. We will assume that the quantity represents the geometric surface area of the protein. It makes sense that proteins with a larger surface area should be more reactive relative to equally massive proteins with a smaller surface area, as chemical reactions take place on the surface of objects. This predictor is calculated using the Shrake–Rupley algorithm. Place a sphere at the $C_{\alpha }$ atom of every AA in the PDB. The radii of these spheres are the same as the radii derived in the Appendix section.

1. Place $N_{i}$ points uniformly on the surface of each sphere *i* (a golden spiral approximation was used to achieve this uniform distribution in our implementation).
2. For each sphere *i*, check all of its $N_{i}$ points to see if any of them lie within the volume of another sphere. If it does, remove that point from the surface of sphere *i*. After this process, there will be $n_{i}$ points remaining on the surface of each sphere, such that $n_{i}/N_{i}$ gives the fraction of the exposed area of the sphere relative to the total area of that sphere.
3. The total surface area of the protein is given by the sum of the fractional surface areas contributed by each sphere to the total surface,
  (A1) $$A=4\pi \Sigma_{i}\frac{n_{i}}{N_{i}}\;r_{i}^{2}$$

Here, *r_i_* is the radius of the sphere of type *i*. The final value for surface area is given in nm^2^.

**Volume:** A brute force check and iteration algorithm were employed to calculate the volume of a protein:

1. Place a sphere at the $C_{\alpha }$ of every AA in the PDB. The radii of these spheres are the same as the radii derived in Appendix B.

2. Place this protein into a box, which itself is divided up into *n* small volume elements of volume ΔV.

3. Iterate through every volume element and count the number of volume elements with centres which lie within the volume of any sphere from the protein.

(A2) $$N=\Sigma_{n}\delta_{nr};\delta_{nr}=\{\begin{array}{c} 1,\;\mathrm{if}\;n\cap r\\ 0,\;\mathrm{if}\;n\backslash r \end{array}$$

4. The total volume of the protein is the number of volume elements counted times the volume of a single volume element.

(A3) $$V=\Sigma_{i}n_{i}\Delta V_{i}$$

The surface area and volume of a protein in and of itself may not be a predictive property, but they can be combined with and enhance other properties in a way that will be discussed in the next sections. The final value for volume is given in nm^3^. This method was used to calculate the volume of all of the proteins considered in the paper when trying to determine the AA radii.

- **Sphericity:** How close a particle resembles a sphere. A highly spherical particle will have a value close to 1. The value is given by
  (A4) $$\Phi =\frac{\pi^{1/3}{\left(6V\right)}^{2/3}}{A}$$
  where *V* and *A* are the protein’s volume and area as previously defined.
- **Surface Area per Mass:** The area of the protein divided by its total mass.
- **Amino Acid count on the surface:** The number of each AA species that appears on the surface of the protein. Being ‘on the surface’ is determined by summing over the surface fractional values from Equation (A1)
  (A5) $$N_{i}=\Sigma_{i}\delta_{ij}\left(\frac{n_{j}}{N_{j}}\right)$$
  where $\delta_{ij}$ is equal to 1 for amino acids of the same type, and 0 otherwise. This methodology is used to calculate the remaining surface properties.
- **Amino Acid percentage on the surface:** The percentage of each AA species that appears on the surface.
- **Amino Acid Dayhoff statistic on the surface:** The percentage of each AA species that appears on the surface, weighted by the Dayhoff statistic.
- **Surface Charge:** The amount of charge that appears on the surface of the protein.
- **Tiny count on surface:** The number of Alanine, Cysteine, Glycine, Serine, and Threonine amino acids on the protein’s surface.
- **Small count on surface:** The number of Alanine, Cysteine, Aspartic Acid, Glycine, Asparagine, Proline, Serine, Tyrosine, and Valine amino acids on the protein’s surface.
- **Aliphatic count on surface:** The number of Alanine, Isoleucine, Leucine, and Valine amino acids on the protein’s surface.
- **Aromatic count on surface:** The number of Phenylalanine, Histidine, Tryptophan, and Tyrosine amino acids on the protein’s surface.
- **Non-Polar count on surface:** The number of Alanine, Cysteine, Phenylalanine, Glycine, Isoleucine, Leucine, Methionine, Proline, Valine, Tryptophan, and Tyrosine amino acids on the protein’s surface.
- **Polar count on surface:** The number of Aspartic Acid, Histidine, Lysine, Asparagine, Glutamine, Arginine, Serine, and Threonine amino acids on the protein’s surface.
- **Charged count on surface:** The number of Aspartic Acid, Glutamic Acid, Histidine, Lysine, and Arginine amino acids on the protein’s surface.
- **Basic count on surface:** The number of Histidine, Lysine and Arginine amino acids on the protein’s surface (positively charged amino acids).
- **Acidic count on surface:** The number of Aspartic Acid and Glutamic Acid amino acids on the protein’s surface (negatively charged amino acids).

## Appendix B

### Evaluation of the AA Radius

One of the input parameters required for our coarse-grained model is the radius of the spherical AA bead. These values determine the volume of the overall protein, which in turn controls the density of the protein. Denser materials tend to have a stronger van der Waals attraction because there is more material present. It is important that the values of the radii chosen preserve the size of the AA and the resulting density of the final protein. We constructed the definition using two measures for AA radius.

The first measure is geometrical. We look at the extreme points of each AA. This is the distance between the centre of mass of the AA and the atom that is the furthest distance away from this point. We called this the etching radius as it is the volume this atom would etch out if it was to rotate and traverse all possible orientations. This radius can be calculated as follows

(A6) $$r_{g}={\left|\vec{\mu }-{\vec{r}}_{i}\right|}_{\max}$$

where $\vec{\mu }$ is the position of the AA centre of mass, and ${\vec{r}}_{i}$ is the position of the *i*-th atom.

The second measure represents the van der Waals radii of the AAs. In a substance consisting of only the particles of interest (in our case a single AA species), the van der Waals radius would be half the average distance to the particle’s nearest neighbour. In this model (and also in our model), the particles are visualized as hard spheres packed together.

These two methods provided very different values for the radii of each AA. The ratio of the radii between these two different methods is mostly constant, meaning that the relative size of each AA is correct, but we need to adjust the scale. This relationship can be seen in Figure A1. This consistency satisfies our first criterion that the AAs should have radii reflective of their relative size. The only outlier is Histidine, which is likely because Histidine is the only charged AA that does not have a long polymer tail. Histidine’s charge would explain its large van der Waals radius as it would strongly repel the nearest neighbour’s, while the lack of a tail would account for the small etch radius.

The next question is to determine which of these two series best represent our model. The second criterion for the choice of radii was that the resulting protein density would match real world values. For this, we refer to [27]. In this paper, the authors measured the mass and density of various proteins and found the following relationship between protein mass and density.

(A7) $$\rho =1.410+0.145\exp\left(-\frac{M}{13.4}\right)$$

where *M* is the mass of the protein. We expect that the density profile of our model proteins will follow this general trend. Using collected protein structure and mass data, it was possible to calculate the density profile for various proteins over a spectrum of different masses. The predicted density profiles from both models are plotted in Figure A2. It is clear from this figure that while both models show the same trend as predicted by Equation (A7), they are off-scale: The etch model is overestimating the density (meaning the radii must be an underestimate), while the opposite is true for $r_{W}$.

To fit the experimental data, we use a linear combination of both data series that gives the desired density profile. This involves minimizing the following function

(A8) $$\rho_{*}=\alpha \rho_{g}+\left(1-\alpha \rho_{W}\right)$$

where *a* is the mixing factor. Carrying out this operation gives a value of *α* = 0.49. A plot of the new density profile derived using this method can be seen in Figure A3. The final set of radii denoted $r_{*}$, which most accurately capture the density and structure, was then calculated as

(A9) $$r_{*}=r_{g}\;r_{W}{\left(\frac{1}{\alpha \left(r_{W}^{3}-r_{g}^{3}\right)+r_{W}^{3}}\right)}^{1/3}$$

The AA radii following from this procedure are shown in Table A1.

The parameters required to calculate the interaction energy between an AA and a NP’s core are the Hamaker constants. This constant of interaction depends on the polarizability of each material and absorption capability. In addition to this, the induced electric field generated by a dipole would also effect, and be affected by, the polarizability of the intervening media between the two materials. As such, the constant that determines the strength of the core potential will depend on the polarizability of three different materials. We have previously calculated these values [10,21] and for convenience we list these values in Table A1.

**Table A1:** Tabulation of AA properties relevant to this work. $r_{*}$ are the optimal AA radii from Equation (A9).

Amino Acid	*n*	$r_{W}$ [nm]	$r_{g}$ [nm]	$r_{*}$ [nm]	$A_{Au-AA}$ [$k_{B}T$]	$A_{Anatase-AA}$ [$k_{B}T$]
ALA	1.606	0.50	0.27	0.32	71.161	7.869
ARG	1.664	0.66	0.35	0.43	72.818	9.466
ASN	1.691	0.57	0.30	0.36	73.556	10.197
ASP	1.700	0.56	0.29	0.36	73.798	10.439
CYS	1.685	0.55	0.29	0.35	73.394	10.035
GLN	1.670	0.60	0.32	0.37	72.984	9.629
GLU	1.655	0.59	0.32	0.38	72.567	9.221
GLY	1.685	0.45	0.24	0.28	73.394	10.035
HIS	1.700	0.45	0.25	0.30	73.798	10.439
ILE	1.568	0.62	0.33	0.40	70.02	6.802
LEU	1.565	0.62	0.33	0.40	69.928	6.717
LYS	1.615	0.64	0.34	0.42	71.425	8.119
MET	1.646	0.64	0.34	0.36	72.314	8.975
PHE	1.682	0.64	0.35	0.33	73.312	9.954
PRO	1.596	0.56	0.30	0.36	70.865	7.59
SER	1.676	0.52	0.27	0.33	73.148	9.792
THR	1.618	0.56	0.30	0.36	71.512	8.202
TRP	1.754	0.68	0.37	0.45	75.204	11.869
TYR	1.643	0.65	0.35	0.42	72.229	8.892
VAL	1.571	0.59	0.31	0.38	70.112	6.887
