# Supplementary figures and images for: In Silico Prediction of Protein Adsorption Energy on Titanium Dioxide and Gold Nanoparticles
Source: Nanomaterials (Basel). 2020 Oct 4;10(10):1967. doi: 10.3390/nano10101967 (PMC7601895; doi:10.3390/nano10101967)

ALA

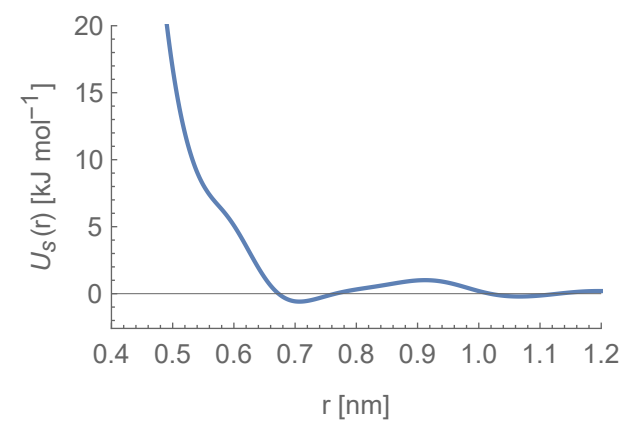

ARG

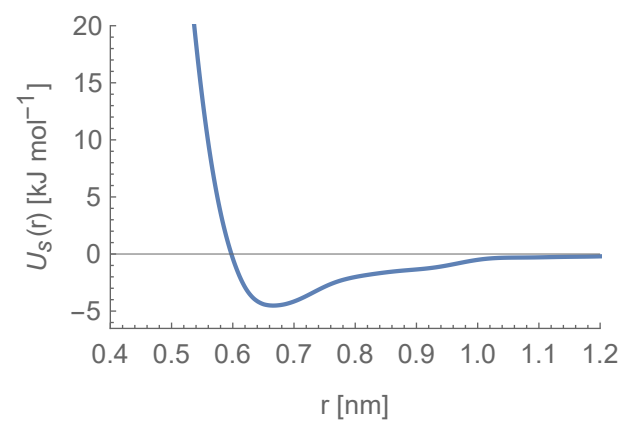

ASN

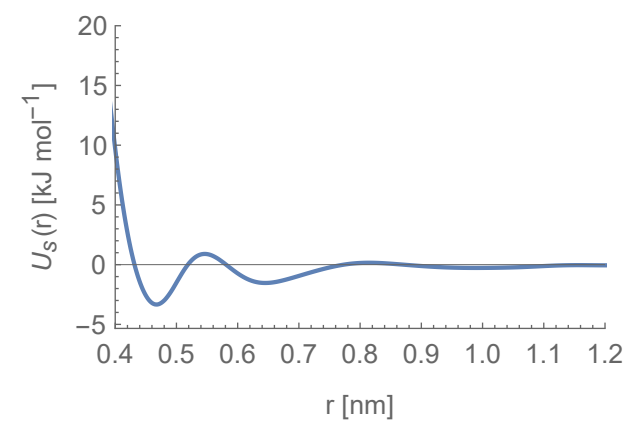

ASP

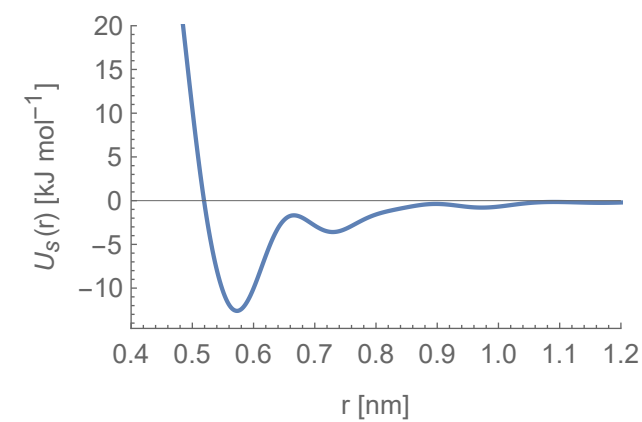

CYS

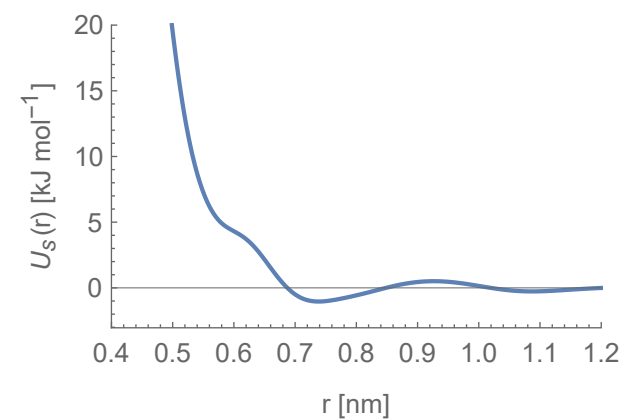

GLN

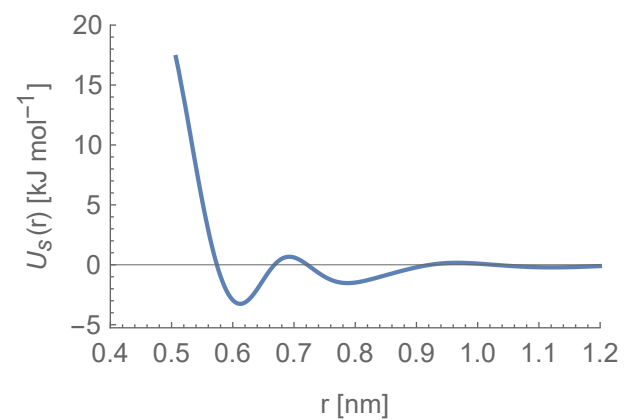

GLU

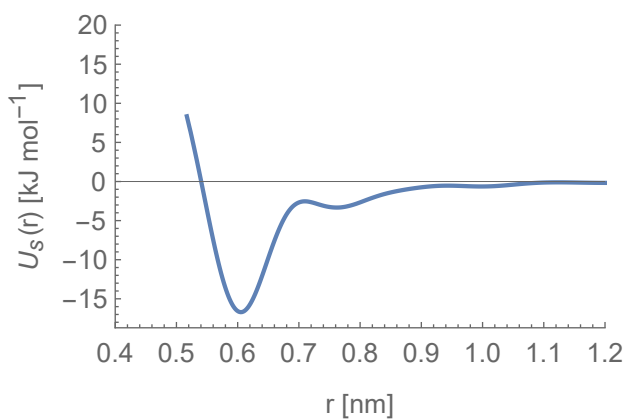

GLY

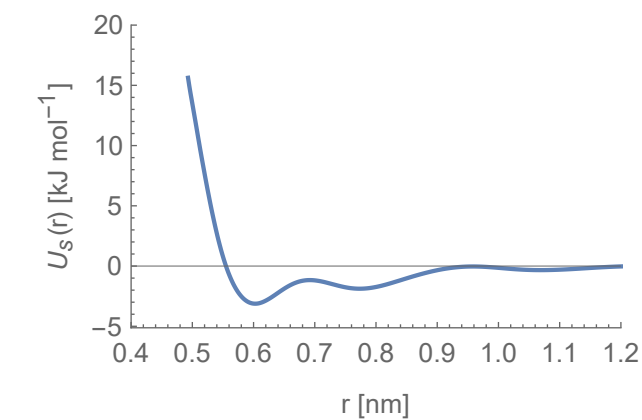

HIS

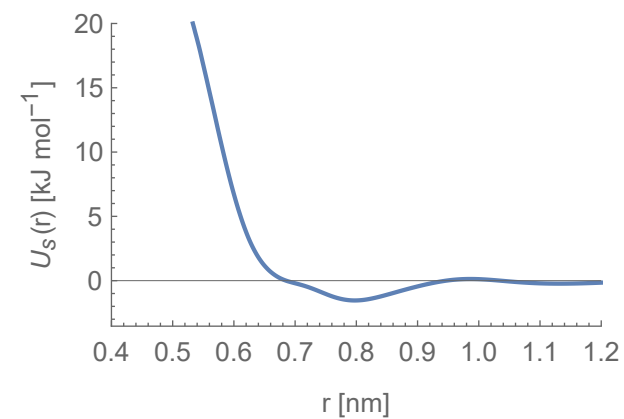

ILE

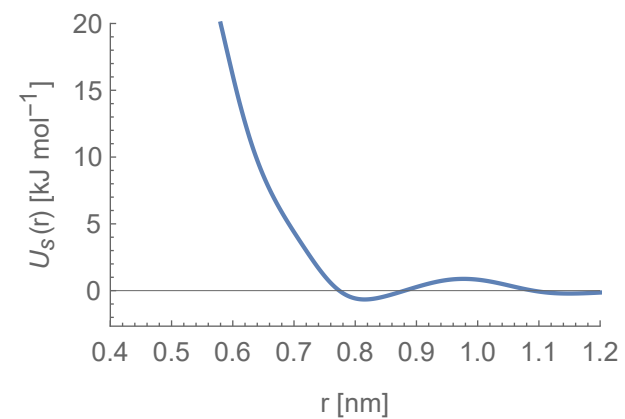

LEU

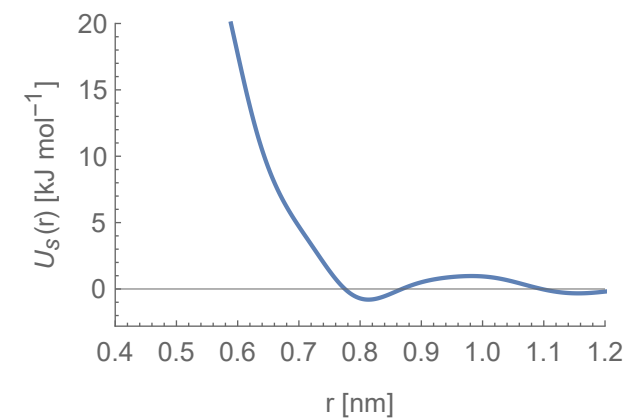

LYS

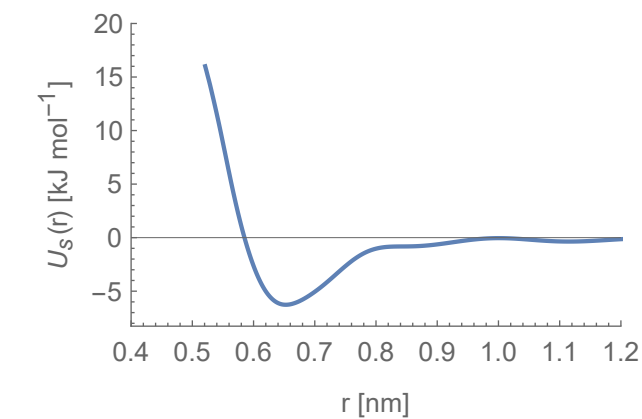

MET

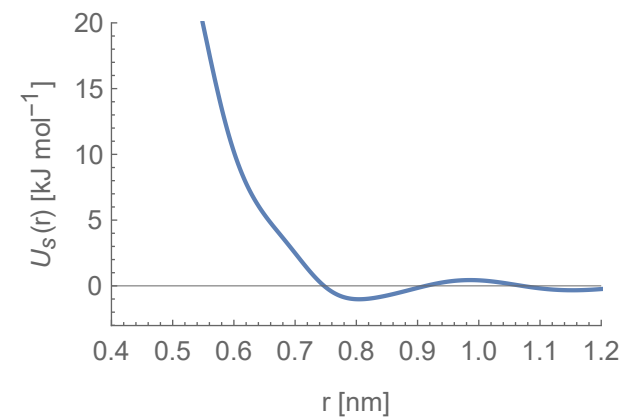

PHE

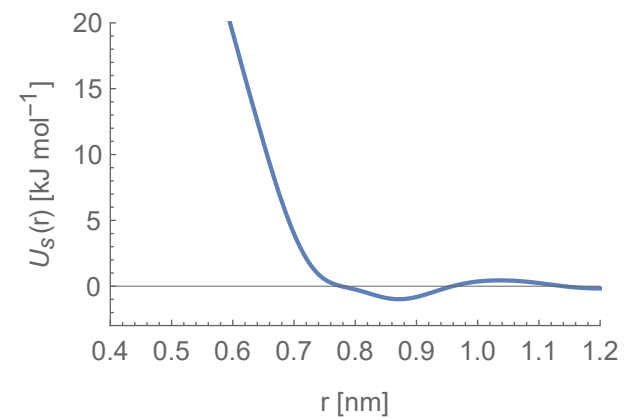

PRO

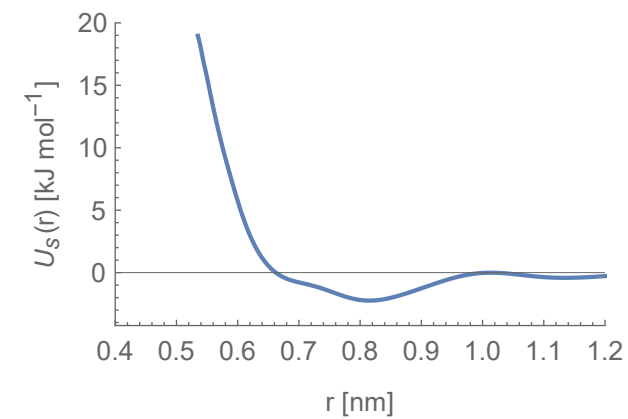

SER

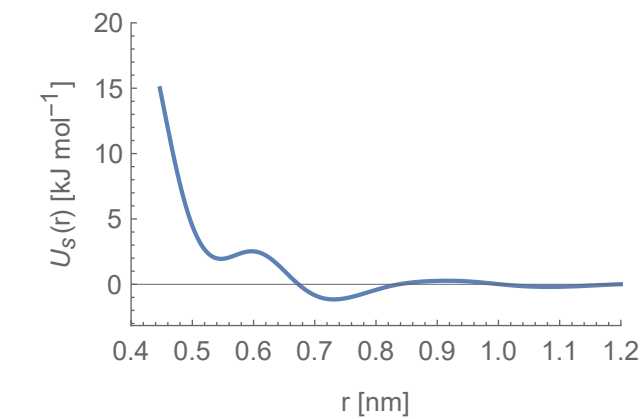

THR

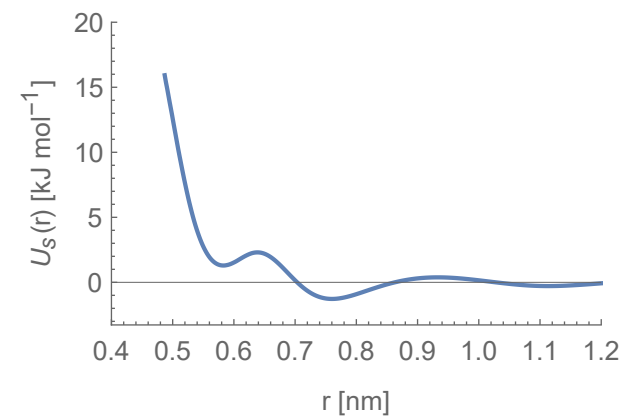

TRP

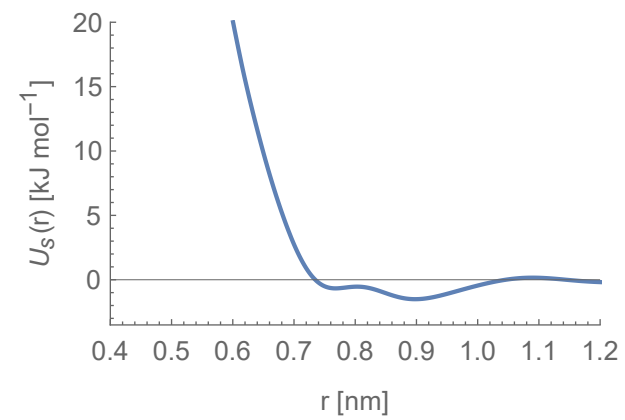

TYR

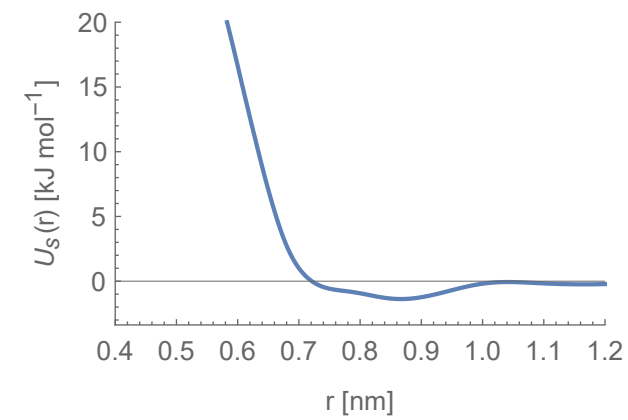

VAL

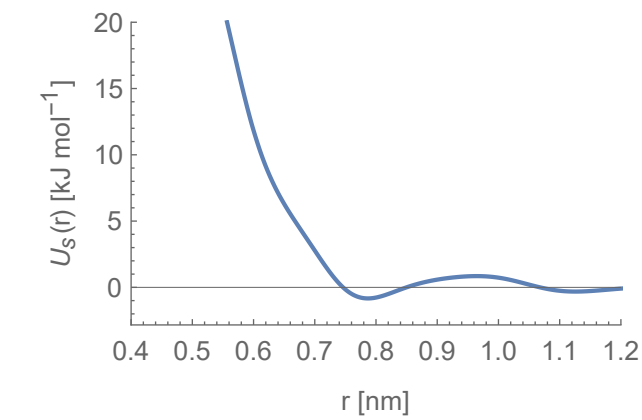

Supplement: Supplementary file 1 [file nanomaterials-10-01967-s001.zip › SI/FigS1.pdf]

ALA

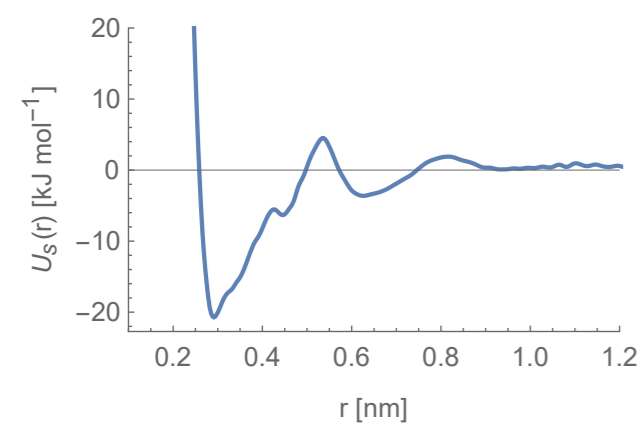

ARG

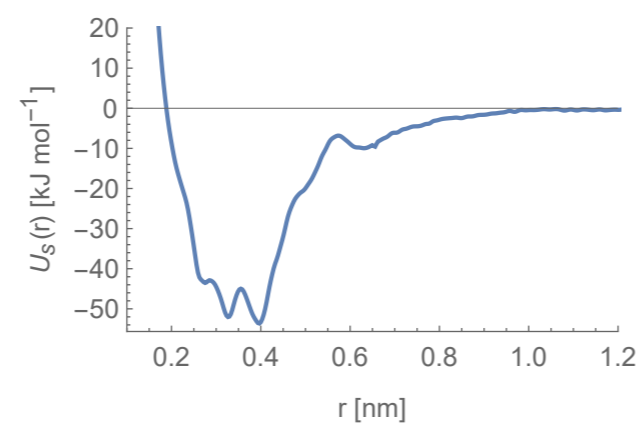

ASN

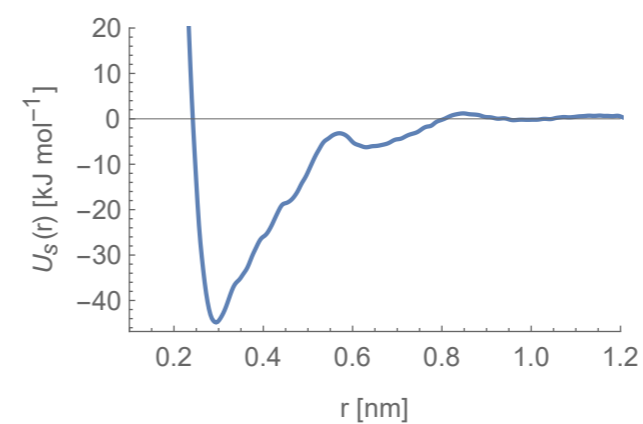

ASPD

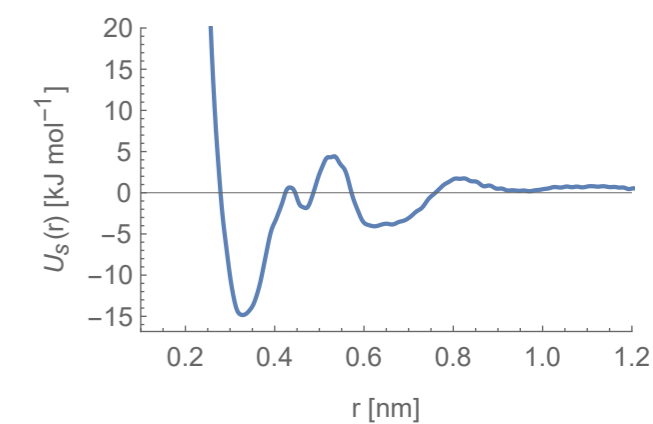

CYS

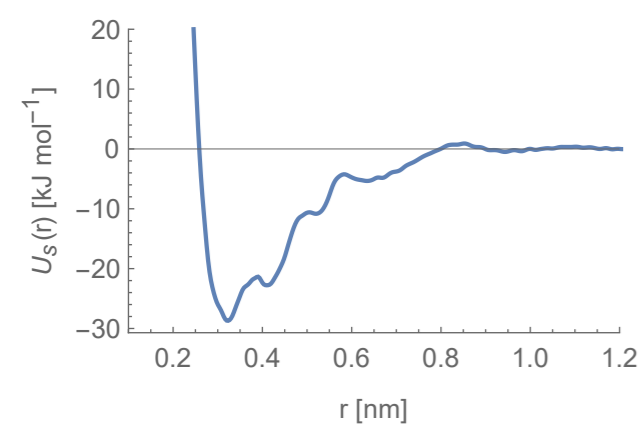

GLN

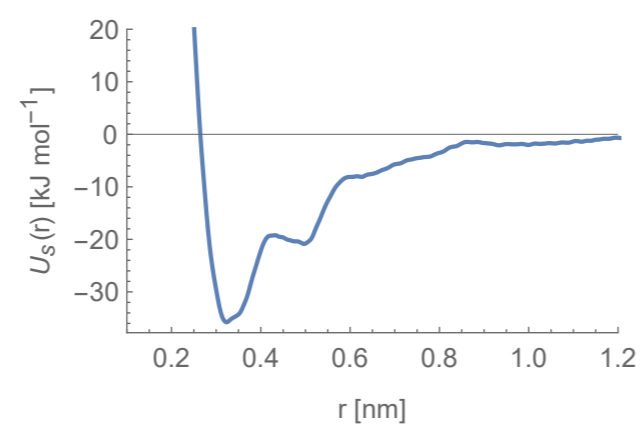

GLU

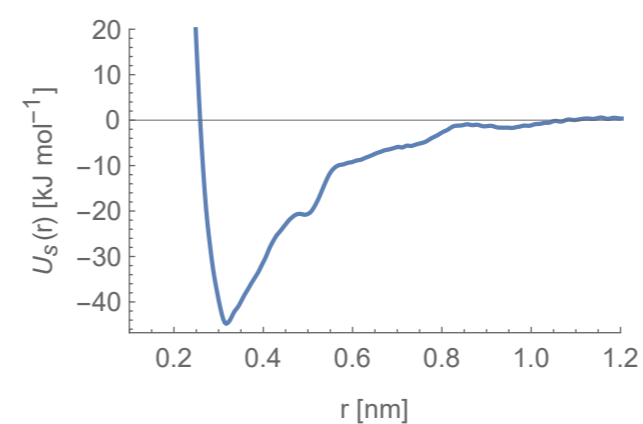

GLY

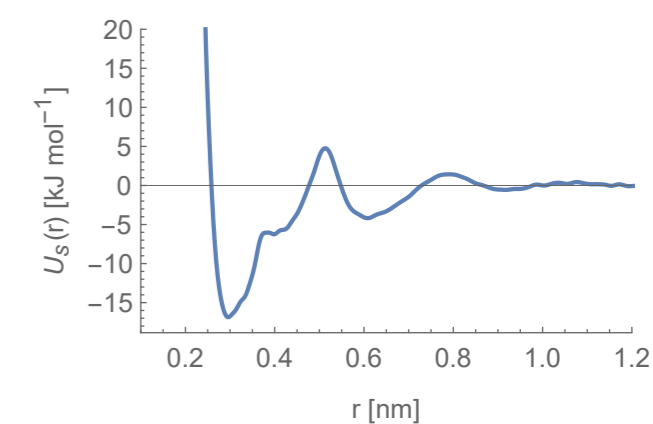

HIS

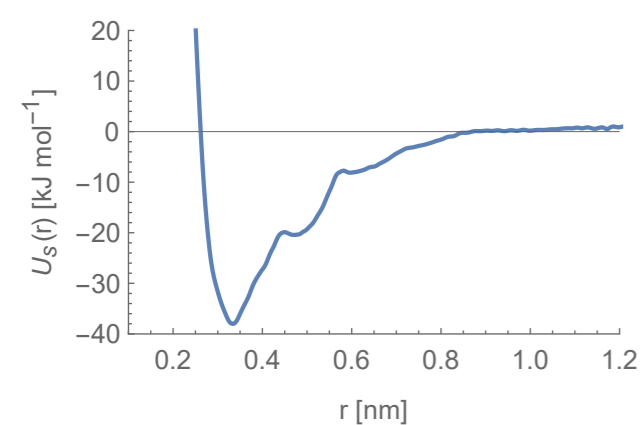

ILE

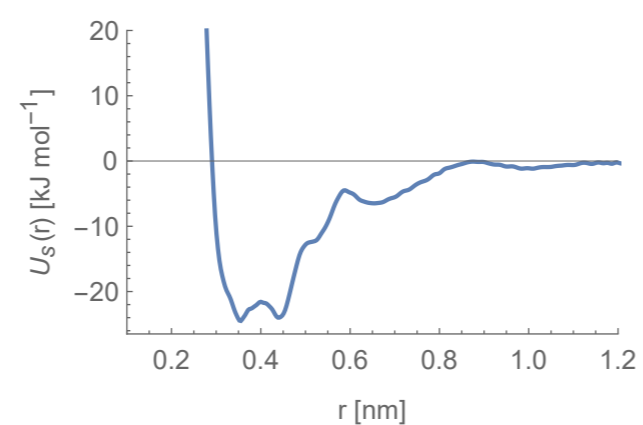

LEU

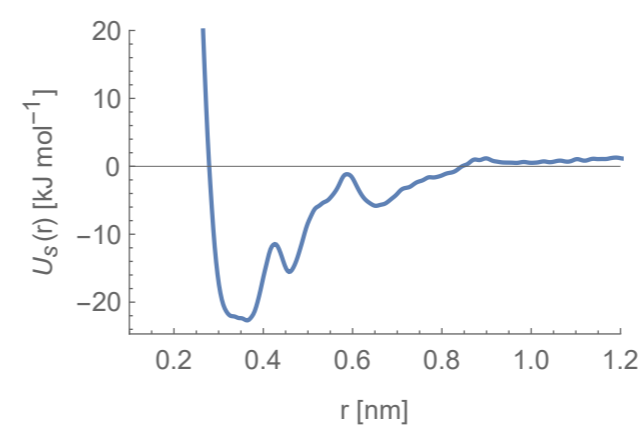

LYS

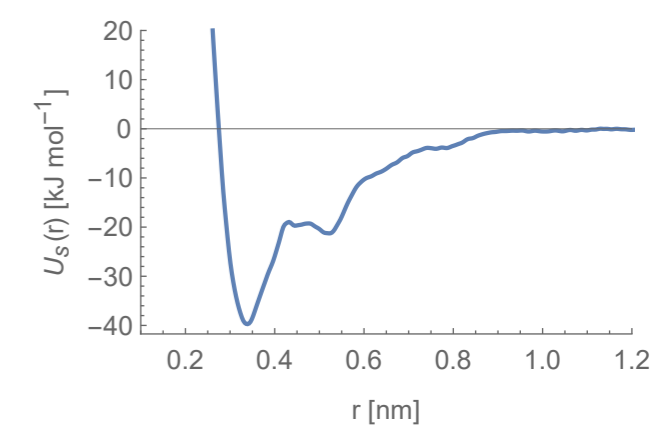

MET

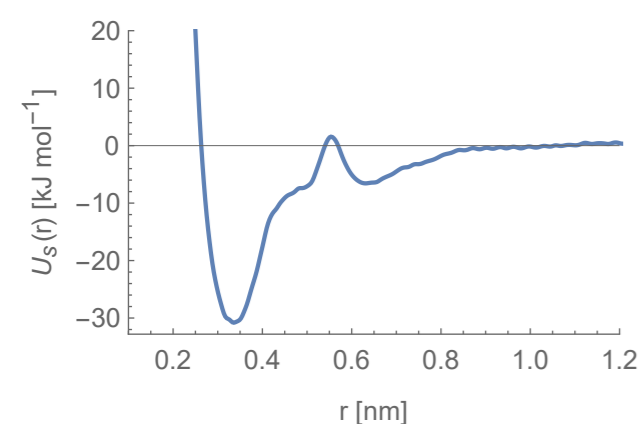

PHE

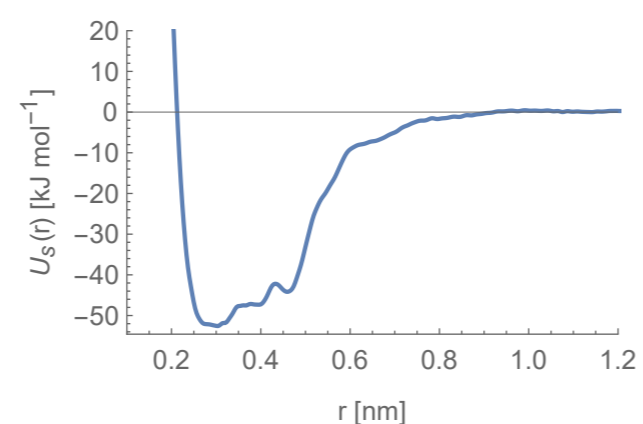

PRO

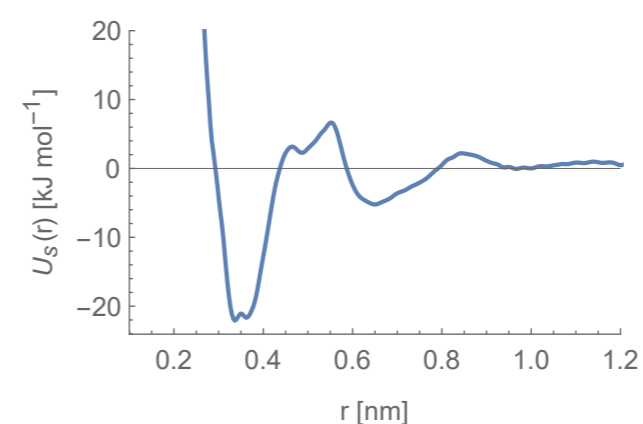

SER

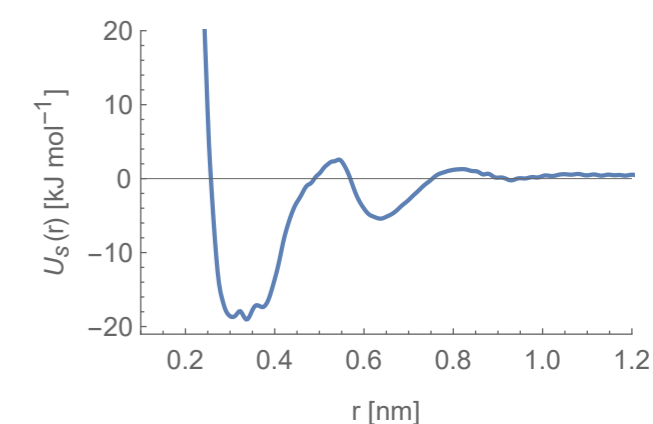

THR

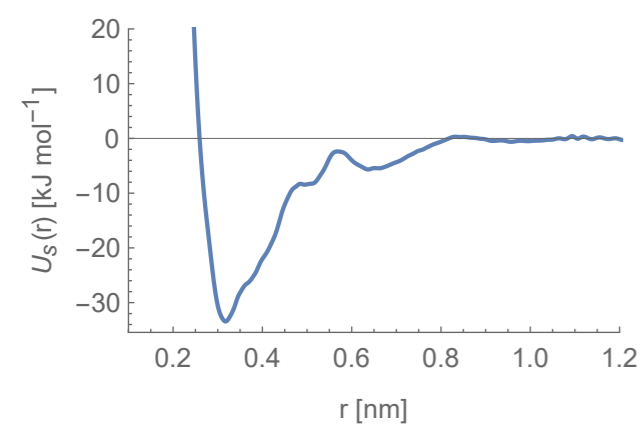

TRP

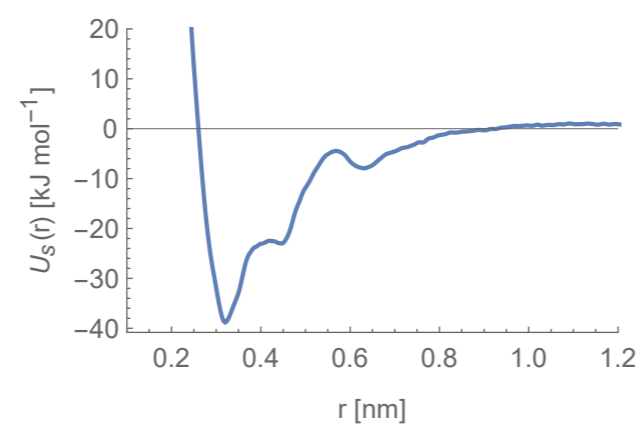

TYR

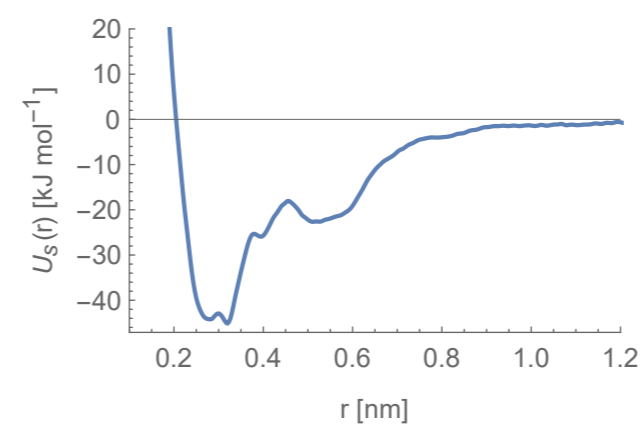

VAL

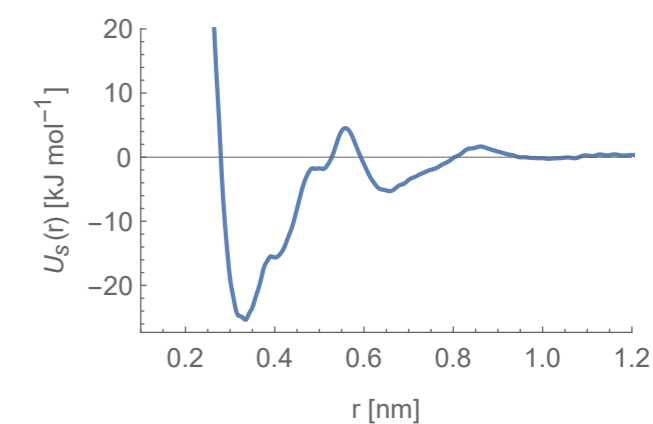

Supplement: Supplementary file 1 [file nanomaterials-10-01967-s001.zip › SI/FigS2.pdf]
